# Agreement between 2 raters’ evaluations of a traditional prosthodontic practical exam integrated with directly observed procedural skills in Egypt

**DOI:** 10.3352/jeehp.2018.15.23

**Published:** 2018-09-27

**Authors:** Ahmed Khalifa Khalifa, Salah Hegazy

**Affiliations:** Department of Prosthodontic, Mansoura Dental School, Mansoura University, Mansoura, Egypt; Hallym University, Korea

**Keywords:** Educational assessment, Dental school, Prosthodontics, Educational measurement, Dental education

## Abstract

**Purpose:**

This study aimed to assess the agreement between 2 raters in evaluations of students on a prosthodontic clinical practical exam integrated with directly observed procedural skills (DOPS).

**Methods:**

A sample of 76 students was monitored by 2 raters to evaluate the process and the final registered maxillomandibular relation for a completely edentulous patient at Mansoura Dental School, Egypt on a practical exam of bachelor’s students from May 15 to June 28, 2017. Each registered relation was evaluated from a total of 60 marks subdivided into 3 score categories: occlusal plane orientation (OPO), vertical dimension registration (VDR), and centric relation registration (CRR). The marks for each category included an assessment of DOPS. The marks of OPO and VDR for both raters were compared using the graph method to measure reliability through Bland and Altman analysis. The reliability of the CRR marks was evaluated by the Krippendorff alpha ratio.

**Results:**

The results revealed highly similar marks between raters for OPO (mean= 18.1 for both raters), with close limits of agreement (0.73 and −0.78). For VDR, the mean marks were close (mean= 17.4 and 17.1 for examiners 1 and 2, respectively), with close limits of agreement (2.7 and −2.2). There was a strong correlation (Krippendorff alpha ratio, 0.92; 95% confidence interval, 0.79– 0.99) between the raters in the evaluation of CRR.

**Conclusion:**

The 2 raters’ evaluation of a clinical traditional practical exam integrated with DOPS showed no significant differences in the evaluations of candidates at the end of a clinical prosthodontic course. The limits of agreement between raters could be optimized by excluding subjective evaluation parameters and complicated cases from the examination procedure.

## Introduction

Diverse evaluation methods are used at dental schools, including practical and written examinations. In addition to objective techniques involving written assessments, clinical assessments should strive to avoid subjective considerations in assessing the clinical competence of examinees. Practical exams are primarily designed to ensure the capability of students to demonstrate the expected attributes and professionalism, communication skills, patient care measures, essential scientific skills, and knowledge required of a dental practitioner [1]. Practical evaluations are likewise diverse, including practical tests in a simulated clinical setting or laboratory, mini-clinical evaluation exercises, directly observed procedural skills (DOPS), objective structured clinical exams, and the traditional practical exam (TPE) [2].

The TPE measures the skills of the students that allow them to solve clinical problems and to perform clinical procedures properly according to evidence-based knowledge. However, the TPE has the drawback of testing a narrow range of skills required for clinical work [3]. The evaluation of DOPS aims to expand the assessment of practical skills in a workplace setting. A student is observed and scored by assessor(s) while performing practical procedures during ordinary clinical work. DOPS can also be included in peer assessments, in which improvements in dental students’ performance can be detected over time [4].

At Mansoura Dental School, the practical clinical exam at the end of the clinical prosthodontic course involves a combination of the TPE and DOPS. As at other dental schools, students must complete a certain number of cases to be eligible to take the practical exam. During the exam session, the patient receives treatment performed by the candidate under supervision. The treatment procedures and the student’s procedural skills are evaluated by 2 raters, and marks are recorded for each student separately. Due to the ubiquitous physiological variation in edentulous patients, different perspectives may exist regarding the most suitable treatment strategy and the most appropriate procedures. This can be reflected in the decision-making of the examined students and their evaluation by the raters, which is why more than one opinion is required.

In this article, we evaluate the agreement of 2 raters’ evaluations of a practical exam that combined the TPE and DOPS at the end of a clinical prosthodontic course.

## Methods

### Ethical statement

This study was conducted on the clinical practical prosthodontic exam administered to pre-graduate students at Mansoura Dental School, Egypt. The study design was approved by the Institutional Review Committee under license number (11030718) after receiving consent from the subjects.

### Study design

This was a descriptive study in which agreement was measured.

### Participants and procedures

**Fig. 1.**
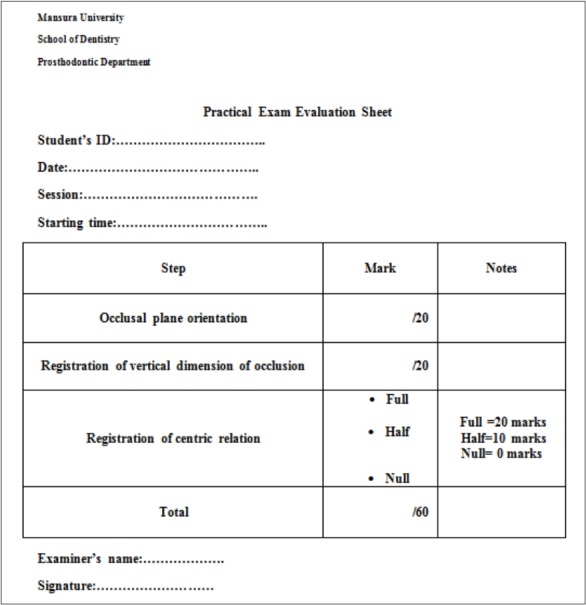
Pre-prepared evaluation sheet.

The study was conducted on a practical exam for bachelor’s degree students from May 15 to June 28, 2017. Students’ identification numbers were recorded in an Microsoft Excel spreadsheet (Microsoft Corp., Redmond, WA, USA) and a sample (n= 76) was selected randomly by computer. The examination procedure involved registering the maxillomandibular relation for a completely edentulous patient, respecting individual physiological variations. The time allowed was 120 minutes. The process was done under a team of supervisors. For the selected sample of students, only 2 raters (AK, SH) monitored the candidates as they worked and evaluated the final registered maxillomandibular relation according to specific guidelines (Supplement 1). Each registered maxillomandibular relation was evaluated based on a total of 60 marks, subdivided into 3 score categories: 20 marks for occlusal plane orientation (OPO), 20 marks for vertical dimension registration (VDR), and 20 marks for centric relation registration (CRR). The marks for each category included a mark for DOPS. The raters reviewed each student’s body language, verbal contact, student-patient position, and handling of materials. The marks assigned by each rater were recorded on separate pre-prepared sheets (Fig. 1). Regarding CRR, students were marked with half of the marks if there was a mistake in evenness of pressure; and with no marks if there was a mistake in both CRR and evenness of pressure. To calculate the final mark for each student, the mean of the marks assigned by both raters was calculated according to the equation:

$$\frac{\left(\mathrm{Total}\;\mathrm{marks}\;\mathrm{of}\;\mathrm{examiner}\;1\;+\;\mathrm{total}\;\mathrm{marks}\;\mathrm{of}\;\mathrm{examiner}\;2\right)}{2}$$

### Statistics

We used the Bland-Altman graph method to measure agreement between both raters in OPO and VDR. As the results for CRR were categorical, we used the Krippendorff alpha ratio. For statistical analysis, IBM SPSS ver. 22 (IBM Corp., Armonk, NY, USA) and MedCalc statistical software (Acacialaan, Belgium) were used.

## Results

**Fig. 2.**
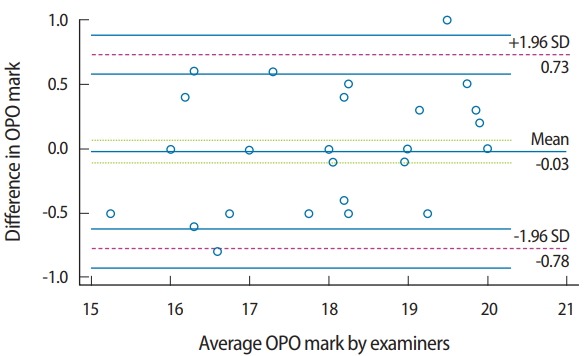
Plots of differences between OPO marks for both raters versus the means of the marks. OPO, occlusal plane orientation; SD, standard deviation.

Identical mean OPO marks were recorded by the raters (mean= 18.1 for both raters), and the mean marks for VDR for both raters were very close (mean= 17.4 and 17.1 for examiner 1 and 2, respectively) (Table 1). The difference in OPO marks for both raters versus the mean values of the marks was plotted (Fig. 2). The mean difference was close to zero (−0.3), with a 95% confidence interval (CI) of −0.113 to 0.0625. The limits of agreement were 0.73 and −0.78. There were 2 outliers, and 50% of the marks (marks for 38 students) were identical between both raters. The plot identified the gap between the limits of agreement as being within 1.5 marks, representing 7.5% of the total score.

**Fig. 3.**
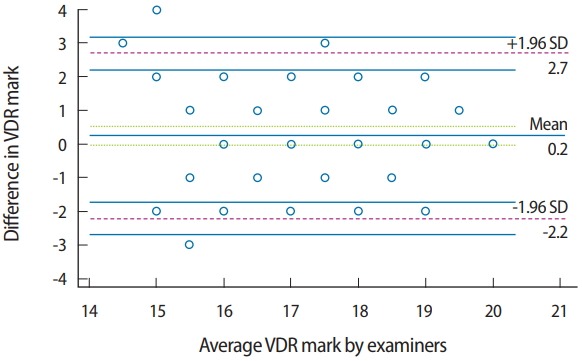
Plots of differences between VDR marks for both raters versus the means of the marks. VDR, vertical dimension registration; SD, standard deviation.

The plots of the difference in the VDR marks assigned by the raters versus the means marks showed a relationship between the absolute difference and the mean. The spread of variables was generally close to the horizontal line representing the mean (0.2; 95% CI, −0.0495 to 0.523), which was close to zero. There were 3 outliers above the upper limit of agreement (2.7) and 1 below the lower limit (−2.2), and 42.1% of the marks (marks for 32 students) were identical between both raters. The plot identified the gap between limits of agreement as being within 4.9 marks representing 24.5% of the total score (Fig. 3).

The CRR marks were categorized into 3 subclasses (null, half, and full marks). Table 2 presents the frequency and percentage of each category for raters. The inter-rater reliability for CRR was assessed by calculating the Krippendorff alpha ratio. There was a strong correlation between the scores assigned by both raters (Krippendorff alpha ratio, 0.92; 95% CI, 0.79–0.99). Raw data are available in Supplement 2.

## Discussion

Dental education programs should strive to teach the proper modalities to treat edentulism [5]. Prosthodontic curricula should aim to equip graduates to manage simple edentulous cases and to recognize patients who require referral to a specialist. At Mansoura Dental School, we teach students in micro-teaching clinical sessions to build the students’ competence to deal with a variety of cases and conditions [6]. In turn, the TPE is designed to test the candidate’s ability to plan and treat patients according to the most suitable approach based on the clinical findings of each patient. We integrated DOPS into the evaluation process as a complementary aspect to augment the weak points of TPE. Additionally, the examination process can be scrutinized by reliability testing to insure its objectivity and to ensure fairness across students.

A graphic representation with a Bland and Altman plot was used to determine reliability by investigating the possible relationship between the discrepancies among the measurements and the true values (proportional bias). The existence of proportional bias is revealed by unequal agreement in the measurements. The limits of agreement are based on the actual measurements. This method is readily understandable and overcomes the artifacts that occur in other reliability tests [7]. The usage of correlation coefficients alone to indicate reliability can be misleading, because the correlation coefficient provides information about the association of variables, but not about proximity or interpretation [8]. The paired t-test can establish whether a difference is significant, in contrast to the correlation test. While the graphic method is the most convenient for continuous variables, the Krippendorff alpha ratio is a highly rigorous measure for assessing inter-rater reliability on rating scales such as those employed in CRR.

The results revealed agreement between the raters for evaluation of the registered OPO. The occlusal plane is a spatial orientation that can be determined by anatomical reference points [9]. These objective characteristics of OPO measurements minimize the variation in orienting the occlusal plane between students and the evaluation by the raters. Another factor enhancing the similarity of OPO among raters and candidates is the method that the students were trained to use to determine the occlusal plane. The candidates were trained to use a ruler or wooden blade to orient the plane in relation to reference points. This method was also used for evaluation by the raters. The outliers in the marks of OPO may be related to differences of opinion between the raters regarding the suitability of the registered treatment plane for the proper esthetics and biomechanics of each patient [10].

The raters’ marks for VDR showed larger error limits than were found for OPO. VDR is not just a measurement between 2 points (an established point on the chin and the other on the tip of the nose), but should be preceded by a proper analysis and understanding of the patient’s condition, about which different perspectives may exist [11]. Such a process may not be feasible given the stress and time limitations of the exam. The instructions given by the student were evaluated, which may have been a source of variation between raters’ marks. Another point regarding the gap in the limits of agreement is the difference in methods used to check the established vertical dimension. While students were trained to use a ruler to measure the distance between reference points, the examiner relied on experience to check the registered vertical dimension based on the esthetic profile of the patient. Both methods are applicable, but the latter is subject to variation [12] and requires more experience, which may make it unsuitable for students. Although many marks were identical between the raters for OPO and VDR, the discrepancies in the VDR marks were noticeable. This can be explained by the subjective parameters of evaluating VDR, as the suitable vertical dimension of occlusion for each patient is determined based on biomechanical considerations that are specific for each patient individually. This led to variation in the DOPS marks for this step. This pattern was not observed for OPO, which depends mainly on objective parameters for evaluation. Thus, we can emphasize that variation among enrolled patients negatively impacted inter-rater reliability.

The raters’ marks for CRR showed a strong correlation. CRR utilizes exact points based on reference lines for verification (the mid and canine lines). This limits the disagreement among raters. Even in different circumstances, trained students can record the centric point. While there is still an indication for more objective methods to standardize the repeated CRR. [13]. Another factor contributing to the strong correlation of CRR marks is the categorical rank (null, half, or full mark) that was used to mark the students in this step. Categorization of marks can mask variation between raters.

Certain other general factors hinder the reliability between raters. The TPE is vulnerable to inter-rater variability, as the examinee faces a real patient with unexpected reactions and more complex problems, which demand more skill than is required for simulated patients. This affects inter-rater reliability, which is sensitive to standardized patients’ characters, patients’ reflections, and raters’ behavior [14]. Examiners may agree or disagree with possible treatment plans based on the physiological condition of each patient. The integration of DOPS marks may also have revealed heterogeneity between raters. The examiners evaluated each candidate based on his/her ability to make correct decisions, to remain poised in stressful situations, and to build rapport with the patient. These factors are subjective and changeable according to the situation and perspective of each examiner, which can affect inter-rater agreement. Other noteworthy factors are the limited time and workplace of the exam, which, in many situations, poses challenges for students’ critical thinking and decision-making [15]. Consequently, we recommend reducing the presence of subjective parameters in evaluation procedures, implementing a unified demonstration of methods for students and for evaluators, and excluding difficult cases that could require different treatment maneuvers from those used in TPE procedures. Ternary evaluation, by the contribution of a third rater, may also improve the fairness and reliability of TPE- and DOPS-based exams.

Within the limitations of this study, including its sample size, we can conclude that the 2 raters’ evaluations of the clinical TPE integrated with DOPS showed reliability for evaluating candidates at the end of a clinical prosthodontic course. The limits of agreement between raters would be further improved by excluding subjective evaluation parameters and complicated cases from the examination procedures. To optimize the TBE- and DOPS-based exam, and to overcome debates regarding its validity, the number of examiners, parameters of evaluation, and standardization of the real patients may be points for further prospective research.

## Figures and Tables

**Table 1.** Descriptive statistics of OPO and VDR marks by examiners

	Examiner 1			Examiner 2		
	Mean	95% CI	SD	Mean	95% CI	SD
OPO	18.1	17.8–18.4	1.2	18.1	17.8–18.4	1.1
VDR	17.4	17.0–17.7	1.4	17.1	16.8–17.5	1.5

OPO, occlusal plane orientation; VDR, vertical dimension registration; CI, confidence interval; SD, standard deviation.

**Table 2.** Frequency of categories for centric relation registration marks by examiners

	Examiner 1	Examiner 2
Null marks (0/20)	3 (3.9)	4 (5.2)
Half marks (10/20)	16 (21.0)	18 (23.6)
Full marks (20/20)	57 (75.0)	54 (71.0)
Total	76 (100.0)	76 (100.0)

Values are presented as frequency (%).

## Supplementary material

Supplement 1. Guidelines for examiners to evaluate the procedure of jaw relation registration

Supplement 2. Data files are available from https://doi.org/10.7910/DVN/8MTQZI.

Supplement 3. Abstract recording.
