# Supplementary material for: Agreement between 2 raters’ evaluations of a traditional prosthodontic practical exam integrated with directly observed procedural skills in Egypt
Source: J Educ Eval Health Prof. 2018 Sep 27;15:23. doi: 10.3352/jeehp.2018.15.23 (PMC6249138; doi:10.3352/jeehp.2018.15.23)
Supplement: Supplementary file 1 — Supplement 1. Guidelines for examiners to evaluate the procedure of jaw relation registration [file jeehp-15-23-suppl1.pdf]

Supplement 1. Guidelines for examiners to evaluate the procedure of jaw relation registration

Procedure	Special parameter of evaluation	General parameter	Marks
Occlusal plane orientation	Height of anterior segment of maxillary and mandibular wax rim related to lips and ridge state according to esthetic and biomechanical factors.<br>Parallelism of anterior segment of maxillary wax rim to the inter-papillary line<br>Height of posterior segment of maxillary wax rim according to biomechanical factors<br>Parallelism of posterior segment of maxillary wax rim to the ala-tragus line<br>Height of posterior segment of mandibular wax rim to 2/3 of retro-molar pad.<br>Centralization of wax rims over the residual alveolar ridge.	Student: patient position<br>Adjusting patient's head in Frankfort plane parallel to the floor<br>Instructions given by the candidate	20
Vertical dimension registration	Proper measuring and registration of vertical dimension of occlusion<br>Suitability of registered vertical dimension of occlusion to the patient's physiological condition.		20
Centric relation registration	Proper registration of centric relation and occlusion of rims together without spaces.<br>Presence of anterior reference lines (mid and canine lines)<br>Homogeneity and evenness of thickness of inter occlusal record material in both sides. Ability to re-check and repositioning blocks intra- and extra-orally.		20
